# Effect of Monthly and Bi-Monthly 50,000 International Units (IU) Maintenance Therapy With Vitamin D3 on Serum Level of 25-Hydroxyvitamin D in Adults: A Randomized Controlled Trial

**DOI:** 10.7759/cureus.13929

**Published:** 2021-03-16

**Authors:** Mohammed A AlAteeq, Abdulaziz AlShail, Amal AlZahrani, Osama AlNafisah, Emad Masuadi, Awad Alshahrani

**Affiliations:** 1 Family Medicine, Ministry of National Guard - Health Affairs, King Abdullah International Medical Research Center (KAIMRC), Riyadh, SAU; 2 Research Unit/Biostatistics, King Saud Bin Abdulaziz University for Health Sciences/King Abdullah International Medical Research Center (KAIMRC), Riyadh, SAU; 3 Endocrinology, College of Medicine, King Saud Bin Abdulaziz University for Health Sciences, Ministry of National Guard - Health Affairs, King Abdullah International Medical Research Center (KAIMRC), Riyadh, SAU

**Keywords:** vitamins, supplements, oral prophylaxis, maintenance

## Abstract

Background

Vitamin D deficiency is a prevalent condition worldwide. Identification of optimal supplementation approaches for maintaining normal vitamin D level in healthy adults is still required. It has been clearly established that sun exposure and diet do not provide the recommended daily amount of vitamin D, and that vitamin D supplementation is needed to maintain normal levels in the Saudi population. The aim of this study was to compare the efficacy of two regimens, monthly and bimonthly doses of 50,000 International Units (IU) cholecalciferol (vitamin D3), in maintaining normal serum levels of 25-hydroxyvitamin D in Saudi adults.

Methods

This study was a randomized controlled trial conducted to compare the efficacy of three regimens in maintaining a normal level of vitamin D in adult individuals. The study took place at three primary healthcare centers in King Abdul-Aziz Medical City, Riyadh, Saudi Arabia. A total of 65 participants were enrolled and randomly divided into two intervention groups and one control group. All participants were contacted by researchers and followed up at their corresponding primary health care center for two successive visits.

Results

All 65 participants completed the study. The participants were mostly females (49; 75.4%); the mean age was 42.1 years (±13.5). The difference in vitamin D levels after three months of the trial was statistically significant among the three groups. A decrease in vitamin D level was recorded in the control group and in the once monthly intake of 50,000 IU group. The bimonthly intake of 50,000 IU group maintained statistically significant vitamin D levels > 75.

Conclusion

Bimonthly vitamin D3 supplementation appears to be an efficient regimen for maintaining a normal level of 25(OH)D, regardless of the amount of vitamin D obtained from diet and sun exposure.

## Introduction

Vitamin D is a fat-soluble vitamin that has an important role in human health, as it plays a major role in regulating calcium and phosphorus metabolism. In addition, it acts at the cellular level in cell proliferation, differentiation, and cell turnover [1]. The bioactive form of vitamin D is a steroid hormone. Vitamin D deficiency affects bone health and other extra-skeletal systems [2-3]. It is defined as a serum level of 25-hydroxyvitamin D (25(OH)D) ≤ 50 nmol/L (20 ng/mL), whereas insufficiency is defined as a serum level of 25(OH)D from 50 to 75 nmol/L [4].

Hypovitaminosis D is associated with metabolic bone disorders, immune regulation, neurogenesis, cardiac diseases, vascular diseases, diabetes mellitus (mainly Type 1), and some types of cancers such as colorectal and breast cancers [5-6]. However, the relationship between vitamin D deficiency and illnesses such as breast cancer and rheumatoid arthritis remains unclear [1].

Vitamin D deficiency is a prevalent condition worldwide. One systematic review conducted in 2018 identified 41 observational studies, which confirmed the high prevalence of clinically silent low levels of vitamin D in the Middle East and North Africa. In that review, vitamin D deficiency prevalence in adults ranged between 44% and 96% with a mean vitamin D serum level ranging from 11 to 20 ng/mL [7]. In Europe and North America, vitamin D deficiency ranges from 20% to 80% in male and female adults [8-9]. In Saudi Arabia, the prevalence of vitamin D deficiency is high. One systematic review of studies conducted from 2011 to 2016 in different age groups showed that the prevalence of hypovitaminosis D (<50 nmol/l) is 81.0%, despite adequate sunlight exposure and diet intake [10-11]. Several factors have been identified as a possible cause of vitamin D deficiency including inadequate sun exposure, low vitamin D intake, and several other physiological factors [12]. Sadat-Ali et al. showed that sun exposure and dietary sources do not provide the daily requirement of vitamin D, and that vitamin D supplementation is needed to maintain normal levels in the Saudi population [13]. This issue was raised before by Hollis who also questioned the adequacy of dietary recommendations in maintaining normal levels of vitamin D [14].

The identification of optimal supplementation approaches for maintaining normal vitamin D levels in healthy adults is still required. In the most recent guidelines on the management of vitamin D deficiency in adults, the Endocrine Society recommends a regimen of once weekly 50,000 IU ergocalciferol or cholecalciferol for eight weeks or 6,000 U/day for eight weeks, followed by a daily dose of 1500-2000 IU as maintenance therapy. However, the duration of maintenance therapy and the effective maintenance therapy for adults with normal baseline vitamin D levels are not clearly defined [2]. The latest guidelines from the American Association of Clinical Endocrinologists recommend maintenance daily doses of vitamin D for certain populations only [15]. In Poland, guidelines for the general population and at-risk groups recommend a regimen of 800-2000 IU/day, tailored individually to body weight, dietary vitamin D intake, sun exposure, and insolation [16]. In the study by Sadat-Ali et al., which included 135 Saudi men and women, the authors found that once serum levels of 25(OH) D normalized, a maintenance dose of 2,000 IU daily for three months was not enough to maintain serum levels of 25(OH)D above 30 ng/mL [13]. Other recommendations suggest that the daily requirement of vitamin D supplementation should range from 800 to 4000 IU/day [4]. An American retrospective chart review study conducted in patients over a period of six years from January 2001 to May 2007 in adults demonstrated that, after completion of the therapeutic regimen, maintenance therapy of 50,000 IU ergocalciferol every other week for up to six years successfully maintained vitamin D levels without adverse effects [17]. Another retrospective study suggested that 50,000 IU vitamin D2 once weekly for eight weeks followed by 50,000 IU every other week for another 3-4 months may be an acceptable maintenance regimen [18]. A prospective study was conducted of the New York Harbor-Healthcare System over a one-year period on commonly used regimens for treating and maintaining normal serum levels of vitamin D. The study included 100 adult patients with a low serum level of vitamin D (<75 nmol/L), who were randomized equally to four groups. The first group received vitamin D3 2,000 IU daily, the second group received vitamin D3 3,000 IU daily, the third group received vitamin D2 50,000 IU weekly, and the fourth group received vitamin D2 50,000 IU twice weekly. The fourth intervention was the most efficacious in achieving and maintaining a 25(OH)D level > 30 mg/mL (75 nmol/L) for 12 months. Notably, the average 25(OH)D level in the fourth group remained >50 ng/mL (125 nmol/L) for nearly nine months, without adverse effects [18].

Little is known in SA about the optimal regimen needed to maintain normal vitamin D levels in adults for longer durations. Thus, the aim of this study was to compare the efficacy of two regimens, monthly and bimonthly doses of 50,000 IU cholecalciferol (vitamin D3), in maintaining normal serum levels of 25(OH)D in Saudi adults.

## Materials and methods

This study was a randomized controlled trial (RCT) conducted to compare the efficacy of three regimens in maintaining normal levels of vitamin D in adult individuals. The study took place at three primary healthcare centers in King Abdul-Aziz Medical City, Riyadh, Saudi Arabia (Health Care Specialty Center, King Abdul-Aziz Housing Clinics, and National Guard Comprehensive Specialized Clinic) from September 2019 to March 2020. All three centers provide primary curative and preventive health services and have both walk-in and appointment scheduling systems for patients to receive treatment and advice for acute and chronic medical conditions.

Participants

The inclusion criteria were: adults aged 18-65 years, both genders, and normal serum level of 25(OH)D (≥30 ng/mL according to the Endocrine Society) at 1 month prior to the study start date [4]. The exclusion criteria were: patients with inflammatory bowel disease, post-bariatric surgery and radiation enteritis, chronic kidney disease, hepatic failure, metabolic bone disease, primary and tertiary hyperparathyroidism, hypercalcemia, active malignancy, autoimmune disease, granuloma-forming disorder (e.g., sarcoidosis, tuberculosis and histoplasmosis), pregnant and lactating women, patient using glucocorticoids, androgen deprivation therapy, anti-seizure medications, immunomodulators and orlistat, alcohol and drug abuse, and mentally incompetent patients. A list of individuals who met the inclusion criteria was obtained from the data center at King Abdullah International Medical Research Center. To detect a minimum difference in the mean Vit D of at least 20 ng/mL between any two groups of the three study groups with a common standard deviation of 20 ng/mL at a 5% level of significance and to achieve a power of 80%, the required sample size found to be 20 patients in each group with a total of 60 patients, who were randomly divided into two intervention groups and one control group. Participants in the first arm were prescribed 50,000 IU cholecalciferol once a month, participants in the second arm were prescribed 50,000 IU cholecalciferol twice a month, and participants in the third arm were the control group (non-intervention group) and did not receive any intervention. Participants were contacted by researchers and followed up at their corresponding primary health care center.

Study design and procedure

The first visit was conducted on September 2019. All participants attended the research clinic to provide written informed consent and fill out the data collection sheet. Then they were randomly assigned to one of the three study arms using a computer-generated number. The data collection sheet contained questions related to demographic data (age, gender, body mass index [BMI], smoking status, comorbidities, sun exposure, calcium-rich food intake). Baseline laboratory data for each study participant were obtained including levels of calcium, magnesium, parathyroid hormone (PTH), creatinine, alkaline phosphatase, and last normal vitamin D level. Each individual in the intervention arms was prescribed 50,000 IU cholecalciferol capsules according to his/her study arm for three months.

The second visit was conducted on December 2019. In this visit, participants filled out the same questionnaire for the second time to reassess calcium-rich food intake, sun exposure, or any new comorbidity that would prevent the patient from continuing the study. Laboratory results were reviewed, and participants’ compliance was assessed. A second set of laboratory data was ordered for each participant for calcium, magnesium, parathyroid hormone, creatinine, alkaline phosphatase, and last normal vitamin D level. At this visit, each individual in the intervention arms was given a refill of 50,000 IU cholecalciferol capsules according to his/her study arm for three months. Subsequent visits were omitted due to the outbreak of COVID-19 and the implementation of a complete curfew during the study course.

Data on 65 participants in the study were entered into IBM SPSS Statistics for Windows, Version 20.0 (IBM Corp., Armonk, NY) for statistical analysis. Descriptive statistics in the form of mean, median, and standard deviation were included. The Student’s t-test was utilized for continuous variables, and the Chi-square and Fisher’s exact tests were used for categorical variables. Analysis of variance (ANOVA) was calculated for multiple groups with continuous variables. The level of significance was set at P < 0.05.

Ethical consideration

Ethical approval was obtained from the institutional review board of King Abdullah International Medical Research Center, with an official memo dated July 24, 2019 (Approval No. RC19/082/R). Data collection sheets were coded in three-digit serial numbers and maintained by the co-investigator. Participants could not be traced after data sheet collection. The study was carried out according to the principles of the Helsinki Declaration.

## Results

Participants and baseline characteristics

**Figure 1:**
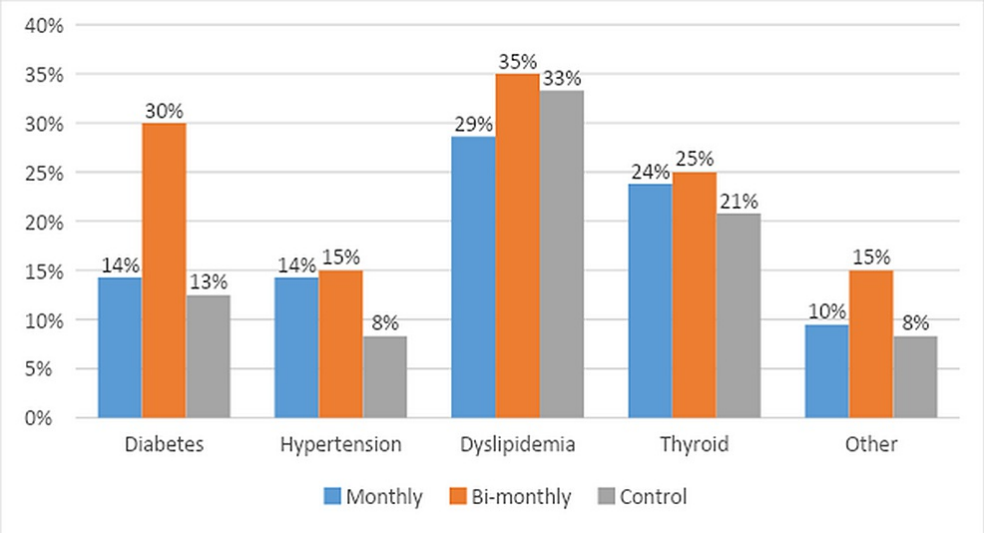
Comorbidities among the three study groups

**Figure 2:**
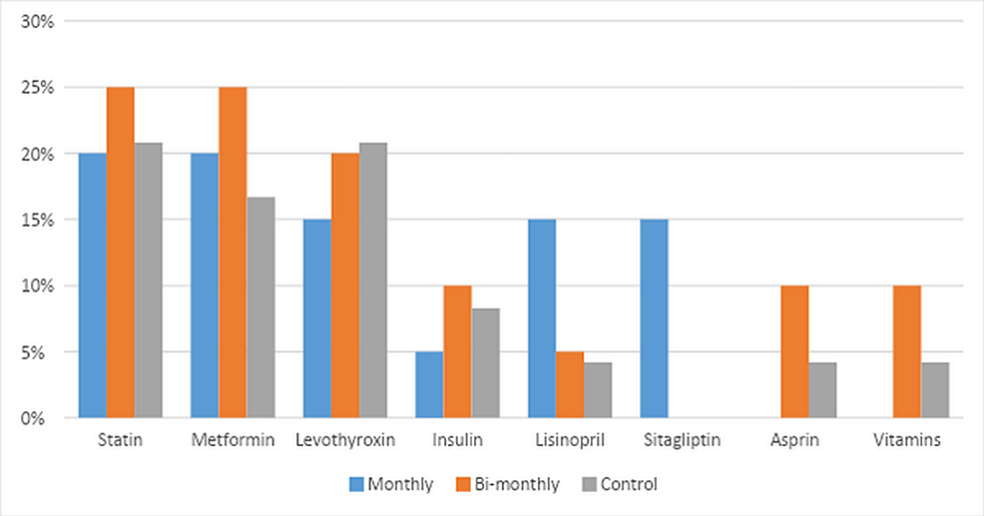
Chronic medication use among the three study groups

A total of 65 participants completed the study. Detailed baseline characteristics, comorbidities, and medication use are illustrated in Table 1 and Table 2, and Figure 1 and Figure 2. The study participants were mostly females (49; 75.4%). The mean age was 42.1 years (±13.5). The most prevalent skin type in the study population was Type 4 by Fitzpatrick classification (36; 58.5%). Participants in all three groups were matched for all baseline characteristics, comorbidities and medication use, with the exception of gender and skin color, which were adjusted for in multivariate regression analysis. Calcium-rich food intake and sun exposure were recorded on a periodic questionnaire throughout the study. All observed differences were statistically significant among the three groups.

**Table 1:** Baseline characteristics of the study groups Based on a: chi-square, b: Fisher’s exact test, and c: ANOVA

Subjects characteristics		Group						Total		P-value
		Monthly		Bimonthly		Control				
		N	%	N	%	N	%	N	%	
Gender	Male	3	14.3	9	45.0	4	16.7	16	24.6	0.059 (a)
	Female	18	85.7	11	55.0	20	83.3	49	75.4	
Body mass index	Underweight	0	0.0	1	5.0	1	4.2	2	3.1	0.795 (b)
	Normal	6	28.6	7	35.0	7	29.2	20	30.8	
	Overweight	5	23.8	3	15.0	8	33.3	16	24.6	
	Obese	10	47.6	9	45.0	8	33.3	27	41.5	
Smoking	No	18	85.7	18	90.0	22	91.7	58	89.2	0.884 (b)
	Yes	3	14.3	2	10.0	2	8.3	7	10.8	
Skin color	T2‎\T3	7	33.3	7	35.0	5	20.8	19	29.2	0.006 (b)
	T4	14	66.7	13	65.0	11	45.8	38	58.5	
	T5‎\T6	0	0.0	0	0.0	8	33.3	8	12.3	
Age (mean±SD)		43±13		46±16		38±12		42.1±13.5		0.137 (c)
Vit D (mean±SD)		100±22.7		97.7±22.9		111.4±27.3		103.5±24.9		0.142 (c)
PTH (mean±SD)		6±2.3		6±2.7		6.7±3.6		6.2±2.9		0.645 (c)
Alk (mean±SD)		68.4±21.6		74.4±20.9		79.3±32.8		74.2±26.3		0.399 (c)
Alb (mean±SD)		44.1±7.4		42.9±2.7		42.1±2.2		43.0±4.8		0.375 (c)
Ca (mean±SD)		2.38±0.09		2.38±0.08		2.36±0.09		2.4±0.1		0.61 (c)
MG (mean±SD)		0.79±0.08		0.8±0.06		0.78±0.07		0.8±0.1		0.618 (c)

**Table 2:** Calcium-rich food intake and sun exposure of the study groups a: Fisher’s exact test

	Group						P-value (a)
	Monthly		Bimonthly		Control		
	N	%	N	%	N	%	
Sun exposure (>5 min)	6	46.2	5	38.5	7	36.8	0.862
Milk intake	9	69.2	12	92.3	15	78.9	0.416
Yogurt intake	9	69.2	9	69.2	11	57.9	0.787
Solid cheeses	4	30.8	6	46.2	8	42.1	0.704
Cream cheese	4	30.8	8	61.5	9	47.4	0.29
Fish or meat	9	69.2	12	92.3	18	94.7	0.148
Sardine	2	15.4	1	7.7	1	5.3	0.805
Egg	10	76.9	10	76.9	17	89.5	0.556

Study outcomes

Differences in vitamin D levels after three months of the trial were statistically significant among the three groups (P-value < 0.001). A decrease in vitamin D level occurred in the control group and in the once monthly intake of 50,000 IU group from 111.4±27.3 to 76.2±21.1 and from 100±22.7 to 89.7±20.4, respectively. However, the bimonthly intake of 50,000 IU group maintained statistically significant vitamin D levels > 75 (97.7±22.9 to 124.5±33.8) (Table 3). Table 4 illustrates the mean vitamin D level after three months of intervention after adjusting for gender and skin color using a general linear model. Using linear model, we found a significant difference between bimonthly and control groups (P-values < 0.001) while no significant difference was found between monthly and control groups (P-values = 0.216).

**Table 3:** Unadjusted mean of vitamin D level after three months of intervention

	Group						ANOVA
	Monthly		Bimonthly		Control		
	Mean	SD	Mean	SD	Mean	SD	P-value
Vit D	89.7	20.4	124.5	33.8	76.2	21.1	< 0.001
PTH	6.4	2.3	6	3	6.2	2.4	0.915
Alk	67.3	21.9	73.6	25.6	77.5	24.9	0.519
Alb	42	2.26	43	2.68	42.24	2.63	0.585
Ca 3	2.34	0.09	2.39	0.05	2.31	0.07	0.028
MG 3	0.8	0.1	0.8	0.1	0.8	0.1	0.874

**Table 4:** Adjusted mean vitamin D level after three months of intervention using a general linear model a: This is t-test for parameters in general linear model *reference group

Variable	Parameter	P-value (a)	Estimate (B)	95% CI for B	
				Lower	Upper
Intercept		< 0.001	74.5	54.9	94.2
Group	Monthly	0.216	12.8	-7.8	33.5
	Bimonthly	<0.001	45.1	22.9	67.3
	Control*	.	0	.	.
Gender	Male	0.893	1.3	-18.7	21.4
	Female*	.	0	.	.
Skin color	T2‎\T3	0.364	12.9	-15.5	41.2
	T4	0.927	-1.1	-26.1	23.8
	T5‎\T6*	.	0	.	.

## Discussion

This is one of the few studies comparing the efficacy of different regimens in maintaining serum level of 25(OH)D in healthy adults. Our study clearly showed that, for individuals with normal baseline 25(OH)D levels, a bimonthly dose of 50,000 IU cholecalciferol is needed to maintain normal serum levels (i.e. ≥30 ng/mL). These findings were observed in the second interventional group, who were given a bimonthly prescription of 50,000 IU cholecalciferol, which resulted in a mean 25(OH)D level of 124.5±33.8 nmol/L.

In a similar but retrospective study conducted by Pietras et al. [17], 86 patients were treated with bimonthly 50,000 IU ergocalciferol (vitamin D2) for a mean of 26 months. The mean 25(OH) D level at the end of the study was 117.3 nmol/L. The mean participant age in that study was 61 years and the mean 25(OH)D level prior to treatment was 58.4 nmol/L, compared to 42.1±13.5 years and 103.5±24.9 nmol/L, respectively, in the current study [17]. In a recent study to establish the quantitative relationship between steady state cholecalciferol input and the resulting serum 25-hydroxycholecalciferol concentration, the total amount from all sources (supplement, food, tissue stores) needed to sustain the starting 25-hydroxycholecalciferol concentration in the winter season for healthy men was estimated at about 3800 IU/d [19], exceeding the bimonthly dose of 50,000 cholecalciferol.

It has been suggested that obese individuals (BMI > 30 kg/m^2^) may require higher maintenance doses of up to three-fold the regular dose [4]. In this study, 41.5% of the participants were obese and 24.6% were overweight. For postmenopausal women, an RCT by Hansen et al. examining treatment with loading doses of 50,000 IU/day daily for two weeks followed by the same dose every two weeks for one year, showed that levels of 200 nmol/L after four weeks and mean levels above 75 nmol/L were maintained for one year [20]. Similarly, for postmenopausal women, an RCT conducted by Aloia et al. showed that maintenance oral dose of 4,000 IU/day produced the highest response [21].

In the current study, the first interventional group given monthly 50,000 IU cholecalciferol had a mean 25(OH)D serum level of 89.7±20.4 nmol/L. These findings clearly indicated a decrease in 25(OH)D levels to suboptimal or deficient levels after monthly 50,000 IU of cholecalciferol for three months. This is consistent with another local study showing that a daily dose of 2,000 IU cholecalciferol (i.e., 60,000 IU cholecalciferol) was insufficient to maintain normal 25(OH)D levels, which significantly dropped to 20.38±5.4 ng/mL [13]. However, these results contradict the 2011 Endocrine Society Clinical Practice Guidelines, which recommend a daily dose of 1500-2000 IU as maintenance therapy in adults from 19 to 70 years of age [4]. They also contradict findings from another prospective study that recruited 100 participants randomized to receive one of four regimens: cholecalciferol (D3) 2,000 international units (IU) daily, D3 3,000 IU daily, ergocalciferol (D2) 50,000 IU weekly, and D2 50,000 IU twice weekly. All groups achieved mean 25(OH)D levels > 30 ng/mL (>75 nmol/L) by five months [18].

It is worth mentioning that, as evidence suggests, cholecalciferol (D3) is more potent in raising and maintaining serum 25(OH)D concentrations, with estimated production of two- to three-fold more storage of vitamin D than ergocalciferol (D2) [22]. This needs to be considered when comparing different studies with different maintenance regimens. The minimum required dose of vitamin D supplements needed to maintain normal serum level of 25(OH)D has been a subject of debate; however, there is no doubt that dietary resources and sun exposure are not sufficient to provide the daily recommended dose of vitamin D. This was confirmed by the current study, which showed that adequate diet and sun exposure were not enough to attain a normal level of 25(OH)D. Sadat-Ali et al. showed that sun exposure and dietary sources did not provide the daily requirement of vitamin D in Saudi individuals [13]. A recent article reviewed food sources over the past decade and found that current dietary habits are not sufficient to maintain a normal level of 25(OH)D, thus underlying vitamin D deficiency in the population [23]. In our study, monthly and bimonthly supplementation of 50,000 cholecalciferol (D3) did not increase calcium serum levels above normal. The mean calcium levels were 2.39, 2.34, and 2.31 mmol/L for the bimonthly and monthly supplementation groups and the control group, respectively. There was no significant difference between the intervention group and control group in the mean serum levels of alkaline phosphatase, albumin parathyroid hormone and magnesium. Hence, bimonthly 50,000 IU vitamin D3 appears to be an acceptable regimen with a reasonable safety profile.

The limitations in this study include the duration, as we were obliged to omit the third visit due to the COVID-19 pandemic and the implementation of a complete curfew at that time. Another limitation was the relatively small study sample as a result of both the difficulty in finding a large number of individuals with a normal level of 25(OH)D and the low response rate, due in part to communication difficulties with participants.

## Conclusions

Bimonthly vitamin D3 supplementation appears to be an efficient regimen for maintaining a normal level of 25(OH)D, regardless of the amount of vitamin D obtained from diet and sun exposure. A large community-based study with large sample is recommended to get strong conclusion in this regard.
